# Live Coverage of Scientific Conferences Using Web Technologies

**DOI:** 10.1371/journal.pcbi.1000563

**Published:** 2010-01-29

**Authors:** Allyson L. Lister, Ruchira S. Datta, Oliver Hofmann, Roland Krause, Michael Kuhn, Bettina Roth, Reinhard Schneider

**Affiliations:** 1 Centre for Integrated Systems Biology of Ageing and Nutrition, Institute for Ageing and Health, Newcastle University, Campus for Ageing and Vitality, Newcastle upon Tyne, United Kingdom; 2 School of Computing Science, Newcastle University, Newcastle upon Tyne, United Kingdom; 3 QB3 Institute, University of California, Berkeley, California, United States of America; 4 Harvard School of Public Health, Department of Biostatistics, Boston, Massachusetts, United States of America; 5 Department of Computer Science, Free University Berlin, Berlin, Germany; 6 Department of Computational Molecular Biology, Max Planck Institute for Molecular Genetics, Berlin, Germany; 7 Biotec, TU Dresden, Dresden, Germany; 8 International Society for Computational Biology, La Jolla, California, United States of America; 9 Computational and Structural Biology Unit, EMBL, Heidelberg, Germany; University of California San Diego, United States of America

## Introduction

Conferences are important hubs of scientific communication, facilitating networking in ways that traditional methods of remote information dissemination cannot match. Internet-based communication is also central to today's science, increasing the accessibility of information and the speed of its dissemination at symposia and conferences. Before live blogging became popular, the best sources of conference coverage were news articles, proceedings, and conversations with attendees. Scientists typically passed relevant information to their local area of influence, while journalists discovered and wrote about connections between presentations, people, and ideas. Now new methods of remote, Web-based communication are augmenting the importance and appeal of conferences by lowering the barrier to scientific communication, as well as increasing the speed with which information is distributed.

The Internet has become instrumental in organizing and advertising conferences. In the past few years, simple Internet-based publishing tools such as blogs have also made it possible for individuals to report and discuss conferences publicly, tasks previously reserved for established media, the organizers, or selected attending scientists. While traditional publishing-house journalism has broadly remained unchanged, many scientists are now publishing their notes on the Internet, accelerating the spread of information to interested audiences. With the increasing popularity of live blogging, conference organizers need to consider how such techniques relate to existing policies. While publication of information at some level is a primary goal of all conferences, there are diverse technological, political, and social factors associated with live blogging that organizers should consider.

Personal homepages and blogs are established centers of scientific communication on the Internet. These have recently been complemented by social networking applications, such as Twitter (http://www.twitter.com) and FriendFeed (http://www.friendfeed.com). Twitter allows microblogging, or the public exchange of short messages of no more than 140 characters, while FriendFeed aggregates and facilitates the discussion of activities across the Web from its users (see Text S1 for an overview of currently available platforms). The emerging interactions of interconnected groups of users via microblogging applications are a form of online social networking more dynamic than blogs or forums due to real time capabilities, simple search and discovery, and low barriers of entry. Scientists who could not attend a talk because of concurrent sessions or because they did not attend in person can still view a live record of what is being presented. In the context of conferences and other events, this real-time reporting is called live blogging. With the advent of live blogging, all conference attendees can become reporters who collect, prepare, and distribute information or related commentary about current events. When presenters are using their talks as a method of publicizing their data, such reporting complements their intentions. Live blogging allows scientists and journalists to have a shared purpose, and as such they should abide by a shared conference reporting policy.

Although blogging and microblogging at conferences has become widespread, policies governing these forms of reporting are rare. A lack of guidance can result in misunderstandings, as was demonstrated at *The Biology of Genomes* conference held at Cold Spring Harbor Laboratory (CSHL) in May 2009. The CSHL policy stated that journalists were to obtain permission from speakers before publishing articles but did not explicitly subject attendees to the same requirement. When Daniel MacArthur of *Genetic Future* blogged and posted comments on Twitter, there were requests for clarification of the CSHL policy [1],[2]. A flurry of news [3] and online discussion followed, both on blogs [4]–[6] and on FriendFeed [7]. In general, science bloggers and journalists felt there should be one policy for all attendees. Most blog posts voiced the opinion that conferences should be as open to these new forms of information sharing as possible.

Responses of organizers to community reporting differ widely. CSHL now obliges everyone to obtain permission from concerned speakers before publishing information on the Web, essentially making community-driven coverage infeasible. In contrast, the International Society for Computational Biology (ISCB) has supported and encouraged live blogging of the Intelligent Systems for Molecular Biology/European Conference on Computational Biology (ISMB/ECCB) 2009 conference[8]. Other conferences, including ISMB 2008 [9], BioSysBio 2009 [10], and the Conference on Research in Computational Molecular Biology (RECOMB) satellite meetings on regulatory genomics and systems biology [11], were covered in a similar fashion.

In the end, live blogging does not change what information is broadcast from a conference, merely how fast it is propagated. This point was made in a letter to *Nature* [12] in response to its editorial recommending that all conferences be either open or closed to live reporting of conference information. Organizers and scientists alike gain from embracing social networking applications, which now support an unprecedented timeliness and level of visibility for both social aspects of the conference and the knowledge presented there. Conferences where information is intended to be public should embrace this timeliness as an amplifier. Other conferences may be better served by more restrictive policies, although presenters should always have the ability to make their presentation public. Whatever decision is made by conference organizers, a clear policy regarding publication of presented information should be advertised. Organizers, attendees, presenters, and journalists all require clear and equitable guidelines. By following the suggestions presented here, conference organizers can shape their policies quickly and simply, and bloggers can provide a useful, timely record of a scientific meeting.

### Openness and Secrecy

The contrast between openness and secrecy in the distribution of information has existed since the time of the ancient Greeks. McMullin contrasts Plato's ideal of “episteme” (knowledge) based on public argument with the mystery religions that restricted knowledge to a few privileged initiates [13]. According to McMullin, “it was [the former] construal of science, as, in principle, open to all that proved the more enduring legacy of ancient Greece,” and it was during the Renaissance when the value placed on originality of thought made it important to obtain proper credit for one's ideas. This evolution led to the emphasis on secrecy in advance of publication.

Information is a nonrivalrous good: if *A* transmits a piece of information to *B*, then *B* is enriched by having the information while *A*'s possession of the information is not diminished. Since the costs of transmitting information are becoming smaller and smaller, it is inevitable that information will flow more quickly and widely. Cooperative enterprises and society as a whole benefit from the free exchange of information. Free software distributions such as the GNU Project (http://www.gnu.org) and the Linux (http://www.linux.org) operating system have contributions from hundreds of volunteers from across the globe. The success of free software has inspired efforts in other realms, such as the translation into English of the original French book on the programming language OCaml, *Développement d'Applications avec Objective Caml* [14], by about 60 volunteers from around the world, communicating solely through the Internet.

A key tenet of science is that it must be possible to replicate results. Conferences are one of the main forums for complete public disclosure of information required for replication. Such publication and advertisement of research is at odds with the need for secrecy in advance of publication to obtain proper credit for one's work. In the life sciences, researchers traditionally keep the results secret until accepted for publication in a peer-reviewed journal, thus establishing precedence. An alternative method of establishing precedence is the dissemination of the information as widely as possible, and as quickly as possible. The physics community has embraced this solution through the arXiv preprint server (http://arxiv.org). It is well understood that this is not a replacement for a peer-reviewed publication, but serves the complementary purposes of dissemination of information and establishment of priority. The lengthy peer review process serves instead to establish that the work meets certain quality standards. Even so, there are other reasons for secrecy: for instance, patents have to be filed before any part of a potential invention can be made public.

Like preprint publishing at arXiv, presenting work at a large conference serves to rapidly disseminate information and establish priority. Dissemination of information by microblogging accelerates and widens distribution beyond the immediate attendees of the presentation, and establishes priority by creating a third-party written record. However, some scientific meetings serve a different purpose. In very specialized fields, it may be difficult to find researchers with similar areas of expertise. Therefore, scientists may travel to small meetings of their colleagues, many of whom may be their competitors. They would rely on the fact that all those present know each other when deciding how much to disclose. Personal trust and responsibility is a major discouragement from so-called “scooping,” and this trust is backed by the presence of a large group of witnesses. While a conference using live blogging lacks the benefits of a small gathering secured by personal trust, it does provide a large group of witnesses and a timestamp for presented work, together with attribution of information to ensure provenance.

## Guidelines for Policy Creation

When a conference is announced, organizers should have an understanding of the subject material to be covered and its suitability for public release. From the beginning, they should develop and advertise fair policies for the presenters and all attendees. Such policies ensure that presenters at an open conference are not surprised when interpretations and discussions of their work are immediately published online, and that scientists and journalists, who are often reporting on the same information, know what is permitted and are treated equally. Conference policy creation is not always easy, and policies generated from these guidelines will help avoid misunderstandings, generate common policies, and enhance scientific communication.

### Guidelines for Organizers

Ideally, organizers of all conferences should create a publishing/blogging policy for attendees early, and advertise it often. Conference organizers need to consider the type of research that will be presented. The style of a conference and the expectations of its audience vary significantly between disciplines. The life science community generally expects presentations on previously published material, whereas computer scientists and physicists expect novel contributions. Conferences such as ISMB, which focus on bioinformatics, often publish full papers of the presentations. What the conference covers will guide the organizers in the creation of a policy. Conferences that highlight published material would be more likely to encourage live blogging, while those covering unpublished results would be less likely to favor such efforts. In determining the policy for their conference, organizers may wish to consider the following:

1. One policy for all types of attendees. Whatever policy is chosen, make it clear that it covers all attendees as, with respect to live reporting of conferences, there is no meaningful distinction between scientists and journalists.
2. Outlawing one medium is not outlawing all. The types of media used by attendees should be considered carefully. Organizers may wish to allow the publication of textual notes, but not photographs or video. Logos have been developed that draw attention to such policy decisions [15]. These can be put on the conference Web site or on presenter slides.
3. Educate your audience. Unfamiliarity with a technology can lead to concerns about its use. When the conference policy is announced, include a short description of what live blogging is and how it is used in conference settings. Focus on what can be gained from the use of such technologies. Awareness of live blogging will also help prevent nonblogging attendees from misinterpreting the typing of a live blogger for Web surfing or emailing, as well as let them know that those bloggers are advertising the presentation rather than ignoring it.
4. Educate session hosts. Suggest that session hosts monitor the microblogging so they can react to feedback from the audience, be it questions from people who are not attending the conference or reports about audio problems.
5. Lower the usage barrier. Ensure that supported technology and tools are prepared well in advance. Use this technology to broadcast announcements and respond to attendee queries. For example, announce your conference's Twitter and Flickr tags (keywords by which interested parties can identify subject-specific posts) and create FriendFeed room(s) ahead of time. At the ISMB/ECCB 2009 conference, the ISCB provided a FriendFeed room that was seeded each morning with presentation names to provide a focal point for note taking. These FriendFeed threads were also embedded into each presentation's ISCB page such that browsing the ISCB site allowed browsing of the real-time presentation discussions.
6. Broadcast your choice early and often. Ensure that whatever policy is agreed upon, it is announced early so speakers and presenters can make an informed decision, enabling them to interact with live bloggers, should they wish to do so.
7. Encourage feedback. Invite speakers to comment on open questions in the blog thread associated with their talk, and solicit feedback from attendees towards the end of the meeting. Be prepared to assist speakers who would like to participate but may be unfamiliar with the microblogging technology.
8. Provide suitable infrastructure. In order to facilitate live coverage of a conference, basic infrastructure is needed at the conference site. A stable and fast wireless network connection in the auditoriums is a must. However, many venues do not yet provide this service, or there may be considerable costs attached to setting up such a service. Ensure a good quality of service for everyone, so conference participants do not disrupt each other. Bloggers also benefit from power outlets that can be used during the talks, as a full day of talks last longer than most computers' battery time.

### Guidelines for Bloggers

A blogger at a conference has a responsibility to follow the policies set out by the conference organizers. The following list of guidelines for conference blogging has been developed to codify efforts that have to date been largely self-imposed, but is not an attempt to legislate the behavior of bloggers at conferences. Bloggers can use these guidelines as aids in determining what limits they wish to impose on themselves.

1. Respect blogging or media policies. Be aware of policies set by the conference organizers and by the presenter of the talk. Conferences provide a medium for both formally announcing work and informally discussing new findings. You might want to have the scientific information available for everyone, but the level of media coverage is the organizers' and speakers' decision to make. When in doubt, approach the organizers beforehand and ask for permission and clarification of their policy.
2. Identify yourself. Consider using your full name or a name that can be linked back to you. Attribution allows others to know who provided notes and commentary.
3. Make a clear separation between personal opinions, questions, and the presentation transcript. If you are covering a talk, readers will expect that most of the text is a transcript of the presentation. Therefore, bloggers should identify any personal comments. Careful consideration should be given to the suitability of blogging personal opinions and remarks that can be picked up during the course of a conference. While we do not suggest a standard way of marking personal comments, microbloggers at ISMB 2009 used simple brackets. Comment indicators from common programming languages (such as “//” or “#”) are also quick and clear. Questions to the presenter from remote or local attendees can be distinguished from the presentation transcript by, for example, prefixing them with a short tag such as “#?.”
4. Focus on the presentation and the science. Disrespect towards the speaker is never appropriate. However, polite criticism on the presented work is appropriate in live blogging and may even stimulate discussions that last beyond the presentation itself. References to related work may prove particularly useful.
5. Use the delete function wisely. It may take a few minutes at the beginning of a well-attended presentation, such as a keynote or plenary talk, to judge how many microbloggers are present. As such, initial comments may be redundant. However, deletion or extensive editing long after the conference violates expectations of timeliness and cooperation.
6. Declare conflicts of interest. If you think that you have a possible bias or conflict of interest, it is polite to make that information available. One possible bias might be a blogger's coverage of a talk by a colleague or friend. Conflicts of interest might exist if a blogger performs research similar to the presenter's.
7. Identify speakers wherever possible. If the presenter is asked questions or if attendees make comments, when capturing those statements it is useful to add the name of person speaking.
8. Ask before blogging on informal conversations. Conferences provide a medium for both formally announcing work and informally discussing new findings. Careful consideration should be given to the suitability of blogging personal opinions and remarks that can be picked up during the course of a conference.

### Guidelines for Presenters

Presenters should keep in mind that the purpose of conferences is dissemination of information. Unless they explicitly agreed otherwise in advance, attendees will naturally spread new knowledge, online or offline. Therefore, presenters should assume that a meeting is open, unless the organizers have stated explicitly in advance that it is closed, such as occurs with many small community meetings. It is the nature of information to spread, so if presenters wish to put limits on the ways in which the information they present can be distributed, it is both their responsibility and prudent practice to make this explicit at the outset. To this end, presenters should review the guidelines below to ensure they are not surprised when they arrive to give their talk.

1. Become familiar with the organizers' specific policies ahead of time, as well as their overall vision of the tone and purpose of the conference.
2. Announce your intentions, if they differ from conference policy. If you wish to be more or less restrictive than the conference policy itself, announce this at the start of your presentation. One way of doing this is through the use of logos such as those proposed by Cameron Neylon [15] to indicate which forms of media are appropriate.
3. Familiarize yourself with the microblogging technology that will be in use at the conference in advance. The microblog can also become a medium for you to advertise your work further by interacting with attendees as well as remote readers. If the posts for the talks are created beforehand, you could also seed the discussion by posting links to the papers and other relevant material.
4. Feel free to contribute to the online debate. Responding to the questions bloggers had during your presentation either in a timely fashion or after a long consideration both have advantages. Responding quickly maintains interest while your talk is still fresh in the attendees' minds.

## Conclusions

Live blogging enhances traditional means of conference coverage by providing a brief public synopsis of a talk and allowing interested researchers to follow up on the presentation, whether by reviewing provided slides, watching a Webcast, or working through relevant publications. In addition to requiring less time and posing fewer technical challenges than a streaming video presentation, live blogging frequently is augmented by thoughtful commentary, direct links to relevant papers, posters, and Web sites. This information is particularly helpful for keynotes, which frequently are not accompanied by a manuscript yet receive strong attendance. Postings by multiple live bloggers ensure coverage from multiple angles. Notes from talks can easily be referenced for follow-up questions via email, searched for keywords and important points, and transferred to more permanent storage locations if required. With microblogging, questions are also opened to a much wider audience, such as those following remotely, and in a much longer time frame.

Completeness and usefulness of conference blogging depends strongly on the presence of attendees willing to participate in live blogging. However, convincing scientists to actively participate can be a challenge. This attitude seems to be at odds with the importance that communication among peers is given at conferences. The reluctance may stem from a lack of knowledge about the technology used rather than an unwillingness to participate, and posts have been written to introduce scientists to these new methods of social networking [16]–[19]. Conference organizers can mitigate these challenges by providing adequate infrastructure, setting clear rules as to what can or cannot be published, advertising the ongoing coverage, and providing information about how to get started microblogging. Presenters can specifically ask for additional questions to be submitted to the blogging area and address these after the meeting, receiving valuable feedback in return. Finally, senior scientists are also encouraged to support their students in their initial forays into social networks as open interaction with other researchers not only provides excellent training, but also opens up venues for exhibiting their own research group to a wider community.

Through the experiences gained at ISMB/ECCB 2009 and other conferences, we have created a set of general live blogging guidelines for conference organizers and participants. These guidelines can help organizers clarify their position on conference reporting as well as inform and reassure attendees. Live blogging enhances scientific communication and, as such, is in keeping with the primary goal of most conferences, which is to broadcast knowledge.

## Supporting Information

Text S1 A summary of current tools for live blogging at conferences, as well as possibilities for the future.
(1.57 MB PDF)
