# Supplementary material for: Live Coverage of Scientific Conferences Using Web Technologies
Source: PLoS Comput Biol. 2010 Jan 29;6(1):e1000563. doi: 10.1371/journal.pcbi.1000563 (PMC2813255; doi:10.1371/journal.pcbi.1000563)
Supplement: Text S1 — A summary of current tools for live blogging at conferences, as well as possibilities for the future. (1.57 MB PDF) [file pcbi.1000563.s001.pdf]

Live Blogging Technology

Current Web Platforms for Live Coverage

A number of platforms are potentially suitable for live coverage of conferences. The most common ones are compared in Supplementary Table S1, and include dedicated services such as CoverItLive (http://www.coveritlive.com/) or ScribbleLive (http://www.scribblelive.com) as well as other more general platforms such as wikis, blogs, Twitter (http://www.twitter.com), FriendFeed (http://www.friendfeed.com) or the upcoming Google Wave (http://wave.google.com). Blogs have been used for a long time to report and reflect on conference talks. Blog posts usually have only one author and are posted after the talk. In contrast, recently emerging services such as Twitter and FriendFeed provide the opportunity for instant coverage of the conference from many angles, broadcast to a world-wide audience.

Supplementary Table S1. Overview of technical solutions for live blogging.

Platform	Pros	Cons
Dedicated conference coverage services (e.g. CoverItLive or ScribbleLive)	• Designed for covering conferences	• High barrier of entry: need separate accounts<br>• Geared towards centralized coverage provided by a dedicated blogging team
Wikis	• Familiar technology<br>• Multiple users	• In practice, only one person can edit at a time<br>• Editing and saving cycles are slow
Twitter	• Low barrier of entry<br>• Provides rapid coverage<br>• Commonly-used as a forum for personal opinions and spontaneous remarks	• 140 characters per message not enough to convey the meaning of scientific talks<br>• Unless special tags ("hashtags") are used, difficult to aggregate
Personal Blogs	• Commonly-used as a forum for personal opinions<br>• Room for detailed commentary	• Difficult to aggregate unless bloggers submit links to their posts to a central location
FriendFeed	• Low barrier of entry<br>• Enables collaborative editing	• Limited format: one heading followed by a long list of plain-text comments
Google Wave	• Enables collaborative editing<br>• Advanced editing features, such as automatic spelling correction<br>• Extensible through 3rd party applications or “robots”, e.g. automatic insertion of chemical diagrams, etc.	• Not released yet

Twitter is a micro-blogging service which allows 140-character long "tweets". While this method of microblogging encourages succinct, direct statements, a single tweet is generally too short to convey the meaning of a scientific talk. Twitter is difficult to use as the main microblogging platform for a conference without overwhelming followers with a multitude of messages.

FriendFeed is a service that enables users to aggregate their "lifestream", i.e. to bundle their posts from sites like blogs, Twitter, bookmarking services and other items of interest from RSS feeds. These bundles are then shared with each user's followers. Many scientists have adopted FriendFeed, focusing on scientific feeds of interest, such as science or technology blogs or papers saved in repositories such as CiteULike (http://www.citeulike.org) and Connotea (http://www.connotea.org). FriendFeed also features "rooms", discussion forums dedicated to a certain topic to which users can subscribe. For example, there is a thriving community of scientists on FriendFeed in the Life Scientists room (http://friendfeed.com/the-life-scientists) and many conferences have created their own rooms. The current use of FriendFeed as a platform to cover conferences began entirely accidentally, as an outcome of the existing community of scientists using the service.

Irrespective of the technology chosen, record permanence is an important unsolved issue. There is no straightforward answer as to how long FriendFeed comments will be stored, and it is noteworthy that FriendFeed was acquired by Facebook in August 2009, making the long-term future of the service as-yet unclear. Twitter does not have an archive, and users are reliant upon third-party tools (http://printyourtwitter.com/, http://twapperkeeper.com/) to keep a longer-term record of what has been said. While conference organizers could strive to create and provide an archive of live blogging efforts, it is unclear if such efforts should be their responsibility.

Future Developments

The ideal conference coverage platform should have a low barrier of entry to allow for efficient collaboration between bloggers. It should also be possible to make room for personal comments and questions next to the more objective transcript of the talk. FriendFeed has currently proven to be the most versatile, but as it only allows for a linear stream of comments without special formatting, it has been mostly used to generate a transcript of the talk. Upcoming developments such as Google Wave merge collaborative editing with information dissemination, document versioning and task automation, effectively combining the power of email, chat-based environments and wikis. Scientists are already working on uses of Google Wave for collaborative research activities (http://blog.openwetware.org/scienceintheopen/2009/08/23/reflecting-on-a-wave-the-demo-at-science-online-london-2009/). Real-time editing should minimize the duplication of content frequently encountered in FriendFeed coverage, as multiple attendants covering the same talk can observe each other's actions, edit existing comments and add corrections as needed. Repository 'robots' (automated participants in a Wave) would allow for the merger of multiple Waves into a coherent summary of a conference, or allow for parallel but connected streams of coverage. A single-threaded comment chain, such as the one shown below in Supplementary Figure S1 from ISMB 2009, could instead be parallelized as shown in Supplementary Figure S2, with one thread displaying factual reporting of the talk and the other covering personal comments and discussion.

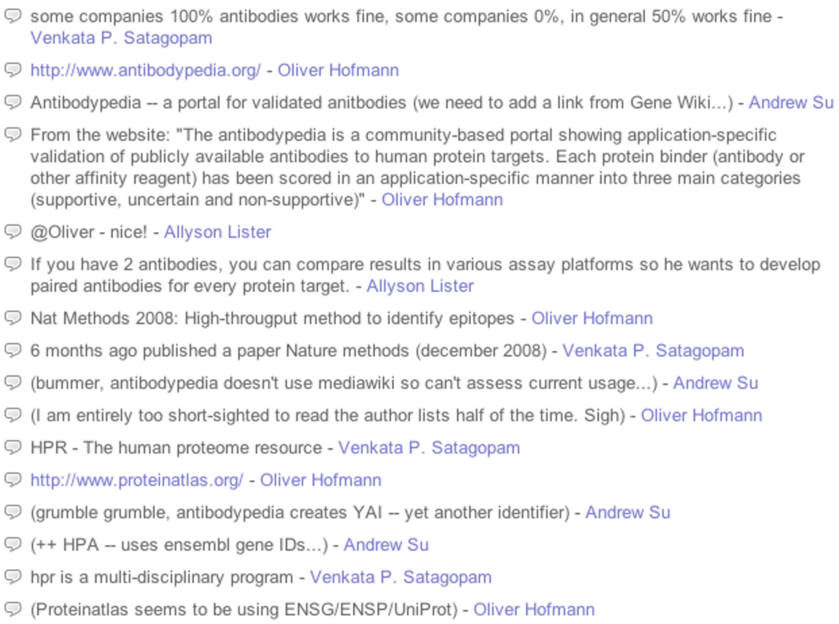

Supplementary Figure S1. A section of the coverage of Mathias Uhlen's keynote at ISMB/ECCB 2009 (http://ff.im/4Hk76).

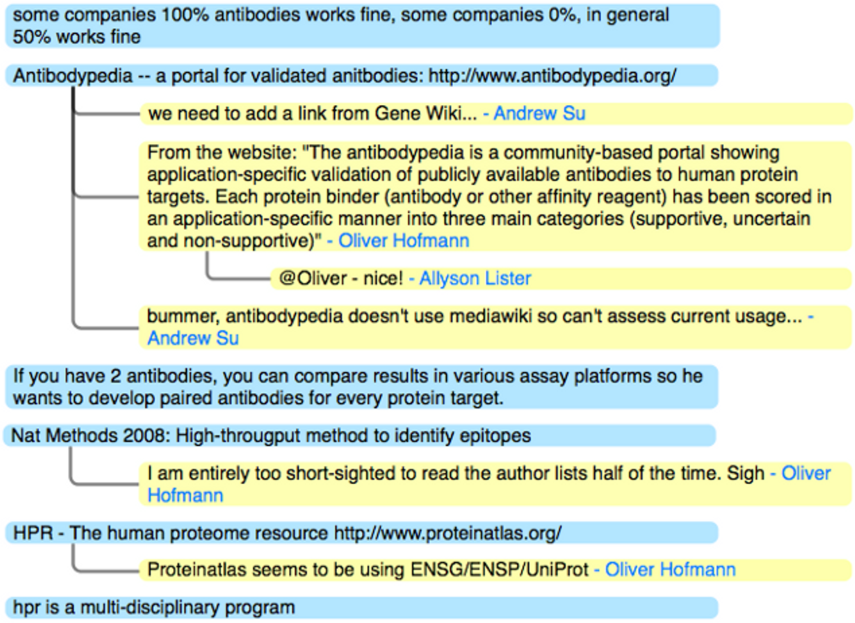

Supplementary Figure S2. A concept for coverage of future conferences based on the same content as Supplementary Figure S1. Here, factual reporting and personal comments are separated yet connected. For the transcript section, author names are hidden, while they are shown for the section containing personal statements. Note that separate posts from Supplementary Figure S1 have been merged, taking advantage of real-time editing facilities.

However, only actual use will determine if such technologies would be too distracting or even work under the conditions of conference network infrastructure. It is noteworthy, however, that the Google Wave protocol is designed to be open and allow multiple servers to offer Waves. Thus, conference organizers could supply their own Wave server within the conference network for faster access times. The success of Google Wave and similar services will ultimately depend on their acceptance within the scientific community, but initial reception has been positive

(http://blog.openwetware.org/scienceintheopen/2009/07/19/sci-bar-foo-etc-part-iii-google-wave-session-at-scifoo, http://network.nature.com/people/mfenner/blog/2009/07/18/using-google-wave-for-a-week-its-still-great) and the first citation manager robots (http://blogs.nature.com/wp/nascent/2009/07/igor_a_google_wave_robot_to_ma.html) are being tested. Additional robots such as those handling automatic semantic annotation of abstracts and commentary (http://groups.google.com/group/knowledge-waves) should be easy to embed and are being actively discussed, but so far lack reported results from actual field tests.
